# Metabolic and blood flow responses to single leg emphasis cycling compared to single and double leg cycling

**DOI:** 10.1113/EP093250

**Published:** 2026-06-10

**Authors:** B. Ryan Davis, Shane Aultman, Safwan Barnawi, Alex Sipolino, John McDaniel

**Affiliations:** ^1^ Exercise Physiology Kent State University Kent Ohio USA; ^2^ Department of Exercise Physiology Louis Stokes Cleveland Veterans Affairs Medical Center Cleveland Ohio USA

**Keywords:** blood flow, carbohydrate oxidation, single leg cycling, small muscle mass

## Abstract

Single leg emphasis cycling (SLEC) has the potential to be beneficial in the rehabilitation setting; however, the metabolic responses to it are unknown. Thus, the current investigation sought to evaluate the metabolic and blood flow responses of SLEC compared to traditional double leg cycling (DLC) and single leg cycling (SLC). This was a within‐participant randomized controlled trial where 12 recreationally active participants completed three different cycling modalities (DLC, SLC and SLEC) at 20%, 35% and 50% of power associated with DLC ${\dot{V}}_{O_{2}\mathrm{peak}}$ (*P*_α_). Gas exchange (${\dot{V}}_{O_{2}}$ and ${\dot{V}}_{CO_{2}}$) was monitored continuously during exercise and rating of perceived exertion (RPE) for the active leg and whole body (WB) was recorded in the last minute of each stage, and blood flow was recorded in the femoral artery immediately following each stage using Doppler ultrasound. Despite similar ${\dot{V}}_{O_{2}}$ across the three conditions, carbohydrate oxidation was greater during SLEC compared to DLC at 20%, 35% and 50% (*P* ≤ 0.047) but, lower than SLC at 35% and 50% *P*_α_ (*P* ≤ 0.011). Similarly, blood flow was greater during SLEC compared to DLC at 20%, 35% and 50% (*P* ≤ 0.010), but lower than SLC at 35% and 50% *P*_α_ (*P* ≤ 0.008). There were no differences in WB or leg RPE between SLEC and DLC across all intensities. The current investigation suggests that SLEC could be an alternative and applicable cycling modality for individuals to increase local metabolic stress for similar levels of exertion and whole‐body metabolic demand.

## Abstract (highlights)

- **What is the central question of this study?**
  Can single leg emphasis cycling create greater localized stress in the emphasized leg compared to double leg cycling at a similar whole‐body metabolic demand?
- **What is the main finding and its importance?**
  Single leg emphasis cycling can create greater localized stress in the emphasized leg for a similar whole body metabolic demand to double leg cycling. The importance is two‐fold: first, it supports the use of single leg emphasis cycling as a training modality; second it can serve as an exercise modality for individuals who may be exercise intolerant due to central cardiovascular limitations.

## 1 INTRODUCTION

Single leg cycling (SLC) is a form of small muscle mass exercise that can be beneficial for both athletic and clinical populations. Acutely, SLC enables the active limb to elicit greater limb specific intensity and blood flow for the same central cardiovascular demand (Burns et al., 2014; Draper et al., 2022; Iannetta et al., 2019). This has been observed in trained cyclist who were able to cycle at supramaximal power outputs during high intensity interval training using SLC compared to double leg cycling (DLC); on average cyclist were able to maintain 15% more limb specific power during single leg cycling (Abbiss et al., 2011). Additionally, people who are limited by central circulation either physiologically or pharmacologically could use SLC to improve exercise related adaptations. For instance, Dolmage and Goldstein (2006) reported that people with chronic obstructive pulmonary disease (COPD) could sustain SLC for a longer duration compared to DLC. Moreover, following 6 weeks of SLC three times per week for 15 min, individuals with COPD had significant improvements in DLC power, 6‐min walk distance, and self‐paced walking (Evans et al., 2015). These findings could be attributed to the adaptations of chronic SLC on muscle metabolic capacity.

Previous investigators have reported differences in substrate oxidation during SLC and DLC, which could be attributed to the increase in peripheral stress observed during SLC. For example, when matched for metabolic and mechanical work, carbohydrate (CHO) oxidation was greater during SLC compared to DLC (Burns et al., 2014; Draper et al., 2019). Draper and colleagues (2019) reported CHO oxidation was 45% greater during SLC at 45% of ${\dot{V}}_{O_{2}\max}$ (SLC 1.46 ± 0.45 g/min compared to DLC 1.01 ± 0.49 g/min). Additionally, previous research has shown significant increases in cytochrome *c* oxidase (COX), a marker of mitochondrial capacity, and glucose transporter (GLUT‐4) following chronic SLC compared to DLC (Abbiss et al., 2011; Dela et al., 1993). Collectively, this allows enhanced ATP synthesis through oxidative phosphorylation.

A drawback to SLC is that it requires some modification to ensure a biomechanically smooth pedalling motion (Elmer et al., 2016), which involves either placing a counterweight on the other pedal or creating a fixed gear bike (Dolmage & Goldstein, 2008; Heidorn et al., 2023). A less studied form of SLC modality is single leg emphasis cycling (SLEC), which is when both legs are moving but one leg is performing the majority of the work. SLEC could be an effective tool for individuals looking for the physiological benefits of SLC without modifying the cycle ergometer and can easily be performed while riding outdoors. Additionally, it could serve as an alternative to SLC to study blood flow regulation and mechanisms of fatigue (Elmer et al., 2013; Stavres et al., 2020). To our knowledge there is only one study that has investigated SLEC. Staples et al. (2020) compared the biomechanics of SLEC to SLC and reported the power produced during SLEC could provide a meaningful training stimulus without the need for specialize equipment necessary for SLC. However, the metabolic responses that occur during SLEC are currently unknown.

Thus, the purpose of this study was to evaluate the effectiveness of SLEC to increase the peripheral stress for the same central metabolic cost as traditional DLC and SLC in individuals who are recreationally active. The primary aim of this investigation was to evaluate the effects of SLEC on CHO oxidation, blood flow and rating of perceived exertion (RPE). We hypothesized that SLEC, similar to SLC, will allow for a greater limb specific power at any given ${\dot{V}}_{O_{2}}$ resulting in a greater peripheral stress for the same metabolic cost as DLC indicated by CHO oxidation, blood flow and RPE.

## 2 METHODS

### 2.1 Ethical approval

This investigation was approved by the Institutional Review Board at Kent State University (IRB no. 1594) and in accordance with the standards set by the *Declaration of Helsinki*. Prior to data collection written informed consent was obtained from all participants.

### 2.2 Protocol

Twelve physically active men (*n* = 8) and women (*n* = 4) volunteered to participate in this investigation (Table 1). Physical activity was self‐reported using the international physical activity questionnaire (IPAQ); all participants reported high levels of physical activity. Then the participants filled out a physical activity readiness questionnaire (PARQ), health history questionnaire (HHQ) and Waterloo footedness questionnaire. If participants did not pass the PARQ or reported any leg injuries, metabolic or cardiovascular disease they were excluded from the study (Table 1).

**TABLE 1:** Participant characteristics.

Measure	Mean ± SD (*n* = 12)
Age (years)	22 ± 5
Height (cm)	171.3 ± 9.3
Weight (kg)	72.6 ± 15.7
${\dot{V}}_{O_{2}\max}$ (ml/kg/min)	38.5 ± 10.2

Participants visited the laboratory on four separate occasions, each separated by a minimum of 24 h. During visit 1, participants performed a 16‐min submaximal cycling protocol followed by a double leg maximal oxygen consumption (${\dot{V}}_{O_{2}\mathrm{peak}}$) test on a Velotron cycle ergometer (Racer Mate, Seattle, WA, USA) while gas exchange was assessed via a metabolic cart (Parvo TrueOne 2400, Sandy, UT, USA). After the ${\dot{V}}_{O_{2}\mathrm{peak}}$ test, a 5‐min familiarization session of SLC was provided for the participants.

During visits 2, 3 and 4, cycling modalities were randomly assigned to each participant; subjects fasted for at least 8 h prior to visiting the laboratory. The protocol for each visit remained the same; the only difference was the cycling modality, DLC, SLC or SLEC. Participants performed a 5‐min warm‐up on the bike at a self‐selected intensity. After the warm‐up participants were provided with 5 min of rest and then fitted with a mask (Hans Rudolph, Shawnee, KS, USA) to monitor gas exchange. Subjects then mounted the bike and performed 5 min of exercise at 20%, 35% and 50% power associated with ${\dot{V}}_{O_{2}\mathrm{peak}}$ (*P*_α_) for a total of 15 min; stages were performed in order from low to high intensity and separated by 3 min of rest. During the last minute of each stage, participants were asked for their RPE using the Borg RPE Scale (6–20) for their whole body and emphasized leg during SLEC (LaScola et al., 2020). In addition, ${\dot{V}}_{O_{2}}$ and ${\dot{V}}_{CO_{2}}$ were recorded throughout the protocol and femoral blood flow was measured immediately at the end of each stage.

### 2.3 Graded exercise test

Initially a 16‐min submaximal test was performed on a Velotron cycle ergometer. Participants were fitted with the mask, and gas exchange was collected on the metabolic cart. The submaximal test was split into four continuous 4‐min stages starting at a low intensity and ending at a moderate intensity. The initial workload ranged from 25 to 100 W across participants depending on their level of physical activity and cycling experience. Each subsequent stage increased by 25 W. Following the submaximal protocol participants were given a minimum of 5 min to recover prior to performing double leg cycling maximal ${\dot{V}}_{O_{2}}$ peak. The ${\dot{V}}_{O_{2}\mathrm{peak}}$ started with 50 W for 3 min and increased 1 W every 2 s until participants reached exhaustion. The ${\dot{V}}_{O_{2}\mathrm{peak}}$ was the highest value calculated using a 30 s rolling average (MacInnis et al., 2017). Maximal effort was determined by a respiratory exchange ratio (RER) ≥ 1.10 and plateau in ${\dot{V}}_{O_{2}}$. The cycling intensities were administered as a percentage of *P*_α_. The *P*_α_ was estimated by plotting the linear relationship between ${\dot{V}}_{O_{2}}$ and power from the submaximal test and extrapolating it to the ${\dot{V}}_{O_{2}\mathrm{peak}}$. This allows for a more accurate estimation of power output to achieve a given ${\dot{V}}_{O_{2}}$ rather than using the power at ${\dot{V}}_{O_{2}\mathrm{peak}}$ during the ${\dot{V}}_{O_{2}\mathrm{peak}}$ test, which is protocol dependent_._

### 2.4 Cycling modalities

During this investigation there were three different cycling modalities, DLC, SLC and SLEC. During each condition participants were asked to cycle between 65 and 70 revolutions per minute (rpm) to maintain the cadence between each visit. During SLC subjects pedalled with one leg while their inactive leg rested on a box next to the ergometer. To ensure a smooth pedalling motion, a counterweight was attached to the unoccupied crank arm (Elmer et al., 2016). If the workload at 20% *P*_α_ was less than 30 W, a 7‐kg mass was used as the counterweight for each intensity; otherwise, a 10 kg mass was used. During the SLEC condition participants were instructed to push down with their dominant leg and de‐emphasize the non‐dominant leg. Participants were given real time visual feedback about the left and right power distribution displayed as a percentage via Velotron Spinscan software. Every minute during the exercise bouts participants were verbally encouraged to focus on the emphasized leg. During SLEC and SLC if participants could not maintain 65–70 rpm the workload was reduced by 5 W. During SLC at 50% *P*_α_, power was reduced by approximately 15 W for two subjects, which allowed them to complete the 5‐min stage.

### 2.5 Substrate utilization

Gas exchange was collected continuously through all three exercise bouts. CHO oxidation was calculated using the last 2 min of each stage using the following equation (Jeukendrup & Wallis, 2005): CHO oxidation (g/min) = (4.344 × ${\dot{V}}_{CO_{2}}$) − (3.061 × ${\dot{V}}_{O_{2}}$)

### 2.6 Blood flow

Heart rate was recorded continuously during exercise using a polar H10 heart rate monitor (Polar Electro, Kempele, Finland); the average heart rate during the last 2 min of each stage was used for data analysis. Prior to exercise with the knee fully extended and leg supported, the Doppler probe (GE Logiq 7, GE Medical Systems, Milwaukee WI, USA) was placed on the common femoral artery 2 cm superior to the bifurcation. The location was marked with a permanent marker for a visual cue to rapidly locate the femoral artery following exercise. Blood flow was measured for 15 s immediately after each stage using a Doppler ultrasound probe. Blood flow was calculated using the standard equation (Wray et al., 2006): Blood flow (ml/min) = [(mean blood velocity) × (π × (vessel radius^2^)) × 60].

### 2.7 Data analysis

The dependent variables in this experiment are ${\dot{V}}_{O_{2}}$, CHO oxidation, blood flow and RPE. ${\dot{V}}_{O_{2}}$, CHO oxidation, RPE and blood flow data were analysed using a two‐way repeated measures analysis of variance (ANOVA). The analysis used three conditions (DLC, SLC, SLEC) and three work rates (20%, 35%, 50%) to determine whether there were any significant main effects or interactions. Data were tested for sphericity using a Greenhouse–Geiser correction. If significant differences occurred, Student's paired samples *t*‐test using a Benjamini–Hochberg *post hoc* analysis was used to determine any differences. Data were analysed using SPSS Statistics software version 29.0 (IBM Corp., Armonk, NY, USA). The data are expressed as means ± standard deviation.

## 3 RESULTS

### 3.1 ${\dot{V}}_{O_{2}}$

**FIGURE 1:**
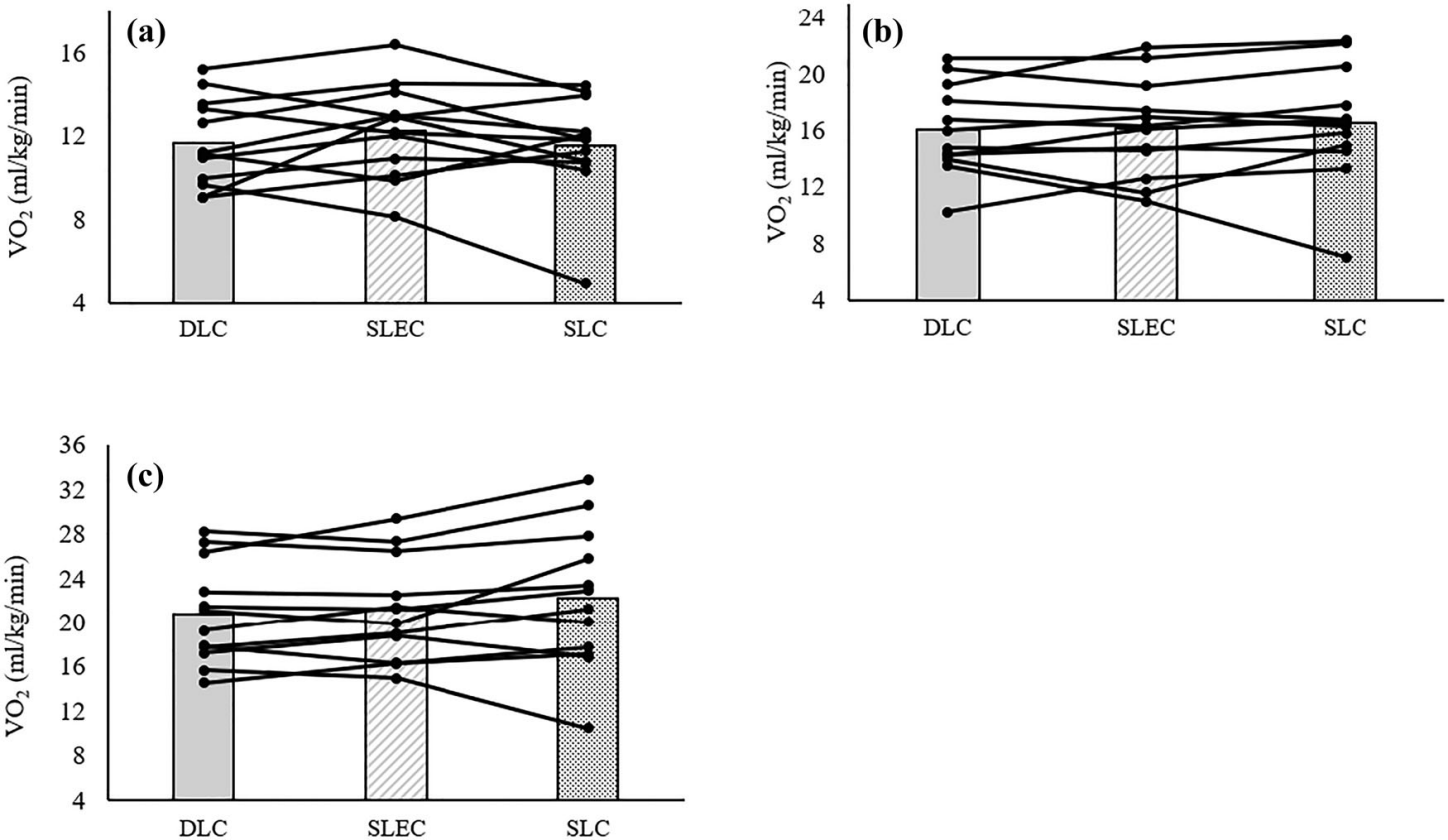
Whole body oxygen consumption during cycling: 20% *P*_α_ (a), 35% *P*_α_ (b), 50% *P*_α_ (c). All comparisons between intensities were significant (*P *< 0.001). However, to focus on the differences between conditions, symbols representing the effect of intensity are not included in the figure (*n* = 12). DLC, double leg cycling; *P*_α_, power associated with ${\dot{V}}_{O_{2}\mathrm{peak}}$; SLC, single leg cycling; SLEC, single leg emphasis cycling.

There was a condition by intensity interaction (*F*(2,22) = 3.661, *P* = 0.047) on ${\dot{V}}_{O_{2}}$. Specifically, ${\dot{V}}_{O_{2}}$ increased with power output as indicated by significant main effect of intensity (*F *= 105.38, *P* < 0.001). ${\dot{V}}_{O_{2}}$ was greater at 50% *P*_α_ compared to 35% *P*_α_ during DLC (*t* = 9.98, *P* < 0.001), SLC (*t* = 7.66, *P* < 0.001) and SLEC (*t* = 13.4, *P* < 0.001). ${\dot{V}}_{O_{2}}$ was also greater at 35% *P*_α_ compared to 20% *P*_α_ during DLC (*t* = 10.46, *P* < 0.001), SLC (*t* = 8.43, *P* < 0.001) and SLEC (*t* = 7.34, *P* < 0.001). However, there was no main effect of ${\dot{V}}_{O_{2}}$ between conditions (*F *= 0.636, *P* = 0.503) (Figure 1). Two subjects’ gas exchange thresholds (GET) were unidentifiable. Of the 10 remaining, GET occurred at 21.95 ± 4.1 mL/kg/min, which was 70% of ${\dot{V}}_{O_{2}\mathrm{peak}}$. During DLC the ${\dot{V}}_{O_{2}}$ at the highest intensity (50% *P*_α_) was approximately 6.87 ± 5.22 mL/kg/min lower than GET.

### 3.2 Substrate oxidation

**FIGURE 2:**
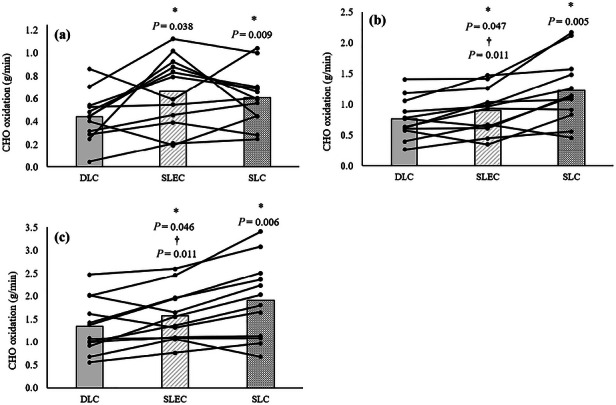
Carbohydrate oxidation across various intensities during cycling: 20% *P*_α_ (a), 35% *P*_α_ (b), 50% *P*_α_ (c). CHO, carbohydrate; DLC, double leg cycling; *P*_α_, power associated with ${\dot{V}}_{O_{2}\mathrm{peak}}$; SLC, single leg cycling; SLEC, single leg emphasis cycling, *Significantly different from DLC; †significantly different from SLC. *n* = 12.

There was significant interaction (*F*(2,22) = 6.703, *P* < 0.001), main effect of condition (*F *= 16.735, *P* < 0.001) and intensity (*F *= 61.555, *P* < 0.001) on CHO oxidation. Specifically, *post hoc* analysis revealed significantly greater CHO oxidation during SLEC compared to DLC at 20% *P*_α_ (*t* = 2.59, *P* = 0.038), 35% *P*_α_ (*t* = 2.30, *P* = 0.047) and 50% *P*_α_ (*t* = 2.39, *P* = 0.046). CHO was not different between SLEC and SLC at 20% *P*_α_ (*t* = 0.73, *P* = 0.479) but CHO oxidation was roughly 22 ± 28% and 12 ± 23% lower during SLEC compared to SLC at 35% *P*_α_ (*t* = 3.54, *P* = 0.011) and 50% *P*_α_ (*t* = 3.44, *P* = 0.011). CHO oxidation was at least 46 ± 42% greater during SLC compared to DLC across all conditions of 20% *P*_α_ (*t *= 6.6, *P* = 0.005), 35% *P*_α_ (*t* = 6.37, *P* = 0.009) and 50% *P*_α_ (*t* = 3.91, *P* = 0.006) (Figure 2). Although no interaction was detected for RER (*F*(2,22) = 2.857, *P* = 0.067), there was a main effect of condition (*F *= 15.633, *P* < 0.001) and intensity (*F *= 62.366, *P* < 0.001). Despite greater CHO oxidation during SLEC compared to DLC across every intensity, a *post hoc* analysis revealed RER was not different between SLEC and DLC at 20% *P*_α_ (*t *= 2.93, *P* = 0.065), 35% *P*_α_ (*t* = 1.813, *P* = 0.109) and 50% *P*_α_ (*t* = 1.875, *P* = 0.113). However, RER was lower during SLEC compared to SLC at 35% *P*_α_ (*t* = 3.310, *P* = 0.013) and 50% *P*_α_ (*t* = 3.687, *P =* 0.009), but not at 20% *P*_α_ (*t* = 0.158, *P* = 0.877) (Table 2). Three participants’ RER value went slightly above 1.0 during SLC at 50% *P*_α_ and one went above 1.0 for SLEC at 50% *P*_α_, which invalidates the equation to estimate CHO oxidation. Thus, for these three participants the ${\dot{V}}_{CO_{2}}$ was reduced so that RER was 1.0 for CHO analysis, which underestimates CHO oxidation.

**TABLE 2:** Measurements of respiratory exchange ratio during cycling.

*P*_α_	DLC	SLEC	SLC
**20%**	0.82 ± 0.04	0.87 ± 0.05	0.87 ± 0.04^*^, (*P *= 0.009)
**35%**	0.85 ± 0.03	0.88 ± 0.05^†^, (*P *= 0.013)	0.93 ± 0.04^*^, (*P *= 0.005)
**50%**	0.91 ± 0.05	0.94 ± 0.03^†^, (*P *= 0.009)	0.98 ± 0.05^*^, (*P *= 0.009)

*Note*: Data expressed as means ± SD. ^*^Significantly different from DLC; ^†^significantly different from SLC. *n* = 12. Abbreviations: DLC, double leg cycling; *P*_α_, power associated with ${\dot{V}}_{O_{2}\mathrm{peak}}$; SLC, single leg cycling; SLEC, single leg emphasis cycling.

### 3.3 Rating of perceived exertion

There was a significant condition by intensity interaction (*F*(2,22) = 8.86, *P* < 0.001) for leg RPE and a significant main effect of condition (*F* = 18.799, *P* < 0.001) and intensity (*F* = 59.71, *P* < 0.001). *Post hoc* analysis revealed that DLC had significantly lower leg RPE values compared to SLC at 35% *P*_α_ (*t* = 4.86, *P* = 0.002) and 50% *P*_α_ (*t* = 5.37, *P* = 0.005) but not at 20% *P*_α_ (*t *= 1.25, *P* = 0.266). Similarly, SLEC resulted in lower leg RPE at 35% *P*_α_ (*t* = 4.71, *P* = 0.003) and 50% *P*_α_ (*t* = 5.48, *P* = 0.009) compared to SLC but there was no difference at 20% *P*_α_ (*t* = 0.00, *P* = 1.00). Additionally, there were no differences in leg specific exertion between DLC and SLEC at 20% *P*_α_ (*t *= 1.25, *P* = 0.303), 35% *P*_α_ (*t* = 0.07, *P* = 0.204) and 50% *P*_α_ (*t* = 2.28, *P* = 0.077). Leg RPE was greater at 35% *P*_α_ compared to 20% *P*_α_ during DLC (*t *= 4.0, *P *= 0.002), SLC (*t *= 5.6, *P *< 0.001) and SLEC (*t *= 4.26, *P *= 0.001). Leg RPE was also greater at 50% *P*_α_ compared to 35% *P*_α_ during DLC (*t =* 3.25, *P *= 0.008), SLC (*t* = 10.9, *P* < 0.001) and SLEC (*t* = 4.3, *P* = 0.001). Whole body RPE was greater at every intensity indicated by a significant interaction (*F*(2,22) = 4.123, *P* = 0.006) and main effect of intensity (*F *= 37.096, *P* < 0.001). Whole body RPE was greater at 35% *P*_α_ compared to 20% *P*_α_ during DLC (*t* = 2.87, *P* = 0.015), SLC (*t *= 4.17, *P* = 0.001) and SLEC (*t* = 2.73, *P* = 0.02). Whole body RPE was also greater at 50% *P*_α_ compared to 35% *P*_α_ during DLC (*t *= 4.71, *P* < 0.001), SLC (*t *= 6.5, *P* < 0.001) and SLEC (*t* = 3.92, *P* = 0.002). However, unlike leg RPE, whole body RPE was not different between conditions (*F *= 2.057, *P* = 0.152) (Table 3).

**TABLE 3:** Perceptive and mechanical responses to cycling.

*P*_α_	Condition	Power (W)	Power dist. (%)	Leg RPE	WB RPE
**20%**	DLC	43 ± 15	45 ± 7	8 ± 2	8 ± 1
	SLEC	45 ± 14	87 ± 11	8 ± 2	7 ± 2
	SLC	44 ± 13	100	8 ± 2	7 ± 2
**35%**	DLC	74 ± 26	48 ± 3	9 ± 2	9 ± 2
	SLEC	74 ± 25	87 ± 10	10 ± 2^†^ (*P *= 0.003)	9 ± 2
	SLC	73 ± 25	100	11 ± 3^*^ (*P *= 0.002)	8 ± 2
**50%**	DLC	105 ± 30	49 ± 4	11 ± 3	10 ± 2
	SLEC	105 ± 36^†^ (*P *= 0.018)	83 ± 10	12 ± 4^†^ (*P *= 0.009)	11 ± 2
	SLC	99 ± 35^*^ (*P *= 0.009)	100	14 ± 3^*^ (*P *= 0.005)	9 ± 2

*Note*: Data expressed as means ± SD. ^*^Significantly different from DLC; ^†^significantly different from SLC. *n* = 12. Abbreviations: dist., distribution; DLC, double leg cycling; *P*_α_, power associated with ${\dot{V}}_{O_{2}\mathrm{peak}}$; RPE, rating of perceived exertion; SLC, single leg cycling; SLEC, single leg emphasis cycling; WB, whole body.

### 3.4 Cardiovascular

**FIGURE 3:**
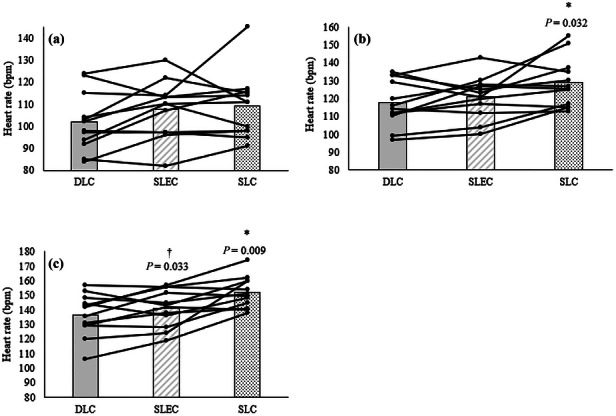
Heart rate response during cycling: 20% *P*_α_ (a), 35% *P*_α_ (b), 50% *P*_α_ (c). *Significantly different from DLC; †significantly different from SLC. *n* = 12. bpm, beats per minute; DLC, double leg cycling; HR, heart rate; *P*_α_, power associated with ${\dot{V}}_{O_{2}\mathrm{peak}}$; SLC, single leg cycling; SLEC, single leg emphasis cycling.

Femoral blood flow demonstrated a sensitivity to both condition and intensity shown by an interaction (*F*(2,22) = 5.244, *P* = 0.002), a significant main effect of condition (*F *= 23.505, *P* < 0.001) and main effect of intensity (*F *= 44.701, *P* < 0.001). *Post hoc* analysis revealed that SLEC resulted in greater blood flow by at least 84 ± 61% compared to DLC at for all three intensities of 20% *P*_α_ (*t *= 3.44, *P* = 0.008), 35% *P*_α_ (*t* = 3.17, *P* = 0.010) and 50% *P*_α_ (*t* = 3.84, *P* = 0.007). Although femoral blood flow was not different between SLEC and SLC at 20% *P*_α_ (*t* = 1.62, *P* = 0.133) SLEC did have 26 ± 22% and 19 ± 14% less femoral blood flow compared to SLC at 35% *P*_α_ (*t* = 3.54, *P* = 0.008) and 50% *P*_α_ (*t* = 3.68, *P* = 0.007), respectively. Blood flow was significantly greater during SLC compared to DLC at 20% *P*_α_ (*t *= 4.35, *P* = 0.003), 35% *P*_α_ (*t* = 5.04, *P* = 0.005) and 50% *P*_α_ (*t* = 4.66, *P* = 0.009) (Table 4). As for heart rate, there was a significant interaction (*F*(2,22) = 214.448, *P* = 0.003), main effect of condition (*F *= 7.900, *P* < 0.001) and intensity (*F *= 156.512, *P* < 0.001). A *post hoc* analysis showed that heart rate was not different between SLEC and DLC at 20% *P*_α_ (*t *= 2.26, *P* = 0.081), 35% *P*_α_ (*t* = 1.26, *P* = 0.263) and 50% *P*_α_ (*t* = 1.81, *P* = 0.126). Moreover, SLEC was not different compared to SLC at 20% *P*_α_ (*t* = 0.48, *P* = 0.644) and 35% *P*_α_ (*t* = 2.32, *P* = 0.092). However, SLEC did result in a lower heart rate at 50% *P*_α_ (*t* = 3.05, *P =* 0.003). SLC did evoked a greater heart rate than DLC at 35% *P*_α_ (*t* = 3.33, *P =* 0.032) and 50% *P*_α_ (*t* = 4.72, *P =* 0.009) but not 20% *P*_α_ (*t *= 2.15, *P* = 0.083) (Figure 3).

**TABLE 4:** Blood flow responses to different cycling modalities.

*P*_α_	DLC (ml/min)	SLEC (ml/min)	SLC (ml/min)
**20%**	1196 ± 449^#^ (*P *= 0.008)	1913 ± 981	2157 ± 1041^*^ (*P *= 0.003)
**35%**	1689 ± 712^#^ (*P *= 0.010)	2683 ± 1246^†^ (*P *= 0.008)	3846 ± 1955^*^ (*P *= 0.005)
**50%**	2381 ± 1005^#^ (*P *= 0.007)	3545 ± 1339^†^ (*P *= 0.007)	4516 ± 1994^*^ (*P *= 0.009)

*Note*: Data expressed as mean ± SD. ^#^Significantly different from SLEC; ^*^significantly different from DLC; ^†^significantly different from SLC. *n* = 12. Abbreviations: DLC, double leg cycling; *P*_α_, power associated with ${\dot{V}}_{O_{2}\mathrm{peak}}$; SLC, single leg cycling; SLEC, single leg emphasis cycling.

## 4 DISCUSSION

To our knowledge this is the first investigation to compare the metabolic responses of SLEC to the more traditional DLC and SLC when matched for absolute power. The results from this investigation partially support our hypothesis. Specifically, despite similar ${\dot{V}}_{O_{2}}$ there was greater CHO oxidation and femoral blood flow during SLEC compared to DLC but less than SLC. Additionally, there were no differences in whole body RPE between cycling modalities. Overall, similar to SLC, SLEC can be used as an additional, potentially more convenient, cycling modality to maximize limb specific intensity and peripheral metabolic stress without additional central cardiovascular stress, study blood flow regulation and induce greater long‐term peripheral adaptations for the same whole body metabolic cost as DLC.

### 4.1 Metabolic

Throughout the three cycling modalities the power distribution between the two legs was split nearly 50/50 during DLC and 85/15 during SLEC while the active leg produced 100% of the power during SLC. Despite these mechanical differences, there were no differences in whole body RPE or oxygen consumption between the three cycling modalities. While there are currently limited data on SLEC, these results agree with previous investigations that also reported no difference in oxygen consumption between SLC and DLC at set power outputs (Burns et al., 2014; LaScola et al., 2020). However, these results disagree with Draper and colleagues (2019) who reported a reduced power output during 30 min of SLC compared to DLC when matched for ${\dot{V}}_{O_{2}}$. Draper et al. reported SLC required less power compared to DLC to evoke the same ${\dot{V}}_{O_{2}}$ during a 30 min bout of exercise, which was likely due to a small ${\dot{V}}_{O_{2}}$ slow component during the extended bout of SLC.

With regards to CHO oxidation, our results indicate SLEC and SLC had greater CHO oxidation than DLC at every workload. Specifically, SLC required 43% greater CHO oxidation compared to DLC. This agrees with Burns and colleagues (2014) as well as LaScola and colleagues (2020) who reported greater CHO oxidation rates during SLC compared to DLC across three absolute power outputs. Similarly, Draper and colleagues (2019) reported a 45% increase in CHO oxidation during SLC compared to DLC despite lower power outputs during SLC. Our data indicate greater CHO oxidation can also be stimulated through SLEC, compared to DLC, at least at the greater intensities utilized in this investigation. This can be explained by the increase in limb specific mechanical work in the emphasized leg, which would lead to a greater metabolic demand contribution from CHO oxidation for ATP. If the chronic adaptations to SLEC are similar to SLC and increase in various metabolic enzymes and transporters including COX and GLUT‐4 expression could be expected (Abbiss et al., 2011).

### 4.2 Cardiovascular

Similar to Burns and colleagues (2014), we observed an increase in blood flow during SLC compared to DLC at every workload. Additionally, SLEC also had greater blood flow than DLC at every intensity but lower blood flow than SLC at 35% and 50% *P*_α_. Thus, there could be benefits with regards to greater muscle perfusion, sheer stress and subsequently upregulation of vascular endothelial growth factor (dela Paz et al., 2012) during SLEC compared to DLC. The greater blood flow during SLC at 35% and 50% *P*_α_, compared to SLEC is likely primarily due to the greater limb specific power production and therefore metabolic demand during SLC compared to SLEC. However, at 20% *P*_α_ the absolute power was lower, and therefore differences in limb specific power between SLC and SLEC might not have been enough to result in a significant difference in blood flow.

The increased limb specific metabolic demand during SLC likely resulted in greater activity from group III/IV afferents (i.e., exercise pressor reflex) ultimately leading to greater a heart rate response (Amann et al., 2010). In healthy individuals the exercise pressor reflex is intensity dependent; higher intensities lead to greater cardiovascular responses (Amann et al., 2010; Sidhu et al., 2019). In the current investigation on average the heart rate during SLC was 11 and 17 beats higher than DLC at 35% and 50% *P*_α_; SLC heart rate was 13 beats/min higher compared SLEC at 50% *P*_α_. These data are similar to findings from Burns et al. (2014) who observed greater heart rates during SLC (9 ± 8 bpm) compared to DLC at their highest reported intensity of 120 W.

### 4.3 Limitations

**FIGURE 4:**
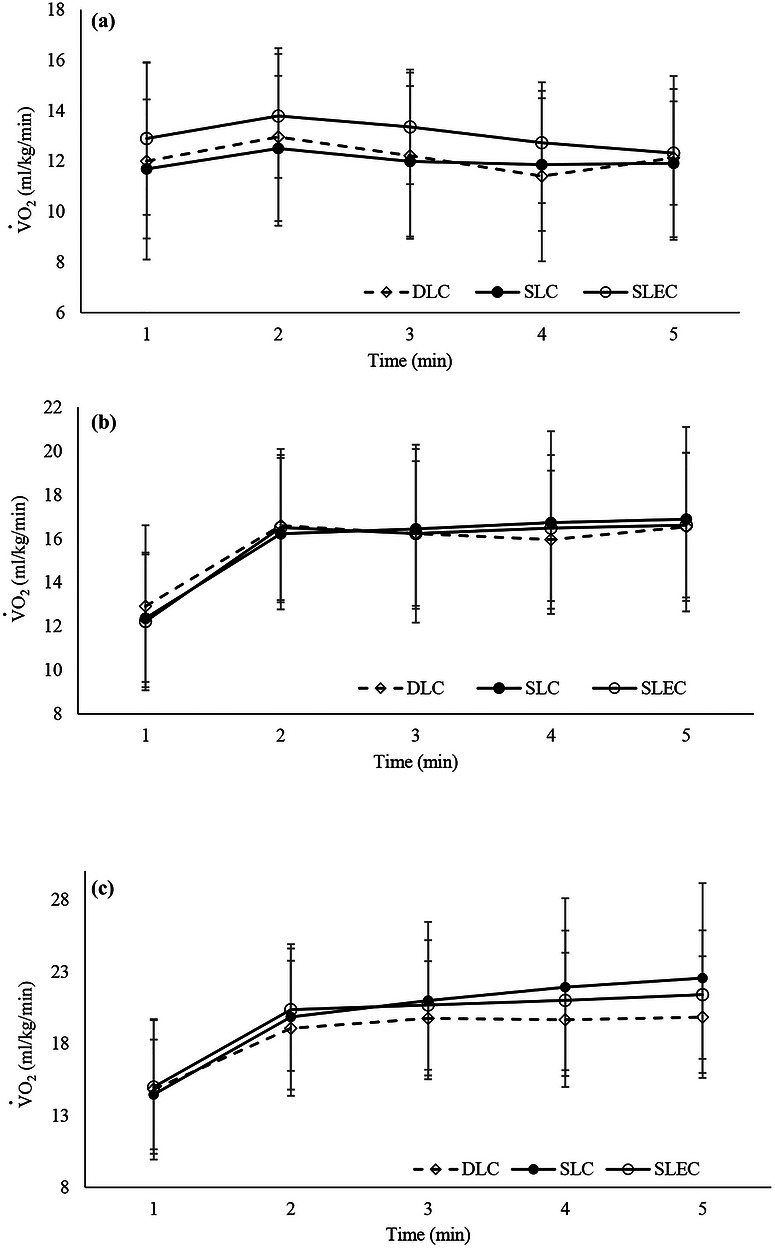
Mean ${\dot{V}}_{O_{2}}$ during each minute of cycling: 20% *P*_α_ (a), 35% *P*_α_ (b), 50% *P*_α_ (c). *n* = 12. DLC, double leg cycling; *P*_α_, power associated with ${\dot{V}}_{O_{2}\mathrm{peak}}$; SLC, single leg cycling; SLEC, single leg emphasis cycling.

As with any investigation, there are limitations within the present study. During SLEC participants cycled at 20%, 35% and 50% *P*_α_. However, in order to maintain the prescribed rpm during SLC at 50% *P*_α_, power output was reduced for two participants, compared to DLC and SLEC. Moreover, given the likely higher relative intensities during SLC and SLEC, it is plausible that a ${\dot{V}}_{O_{2}}$ slow component developed during the 5‐min stages, particularly at 50% *P*_α_, which may have influenced substrate oxidation estimates. However, we believe that these effects are minimal (Figure 4). Additionally, every participant reported that SLEC required significantly more focus and attention compared to DLC and SLC.

### 4.4 Conclusion

Our primary objective was to observe the metabolic effects of SLEC compared to DLC and SLC. We found that SLEC results in greater CHO oxidation and limb blood flow compared to traditional DLC for the same whole body ${\dot{V}}_{O_{2}}$ and RPE. Although the increases in CHO oxidation and blood flow during SLEC were not as great as during SLC, the trade‐off would be the ability to perform SLEC without modifying a cycle ergometer and being able to perform it while cycling overground. This investigation demonstrated that SLEC can be used as an alternative to SLC to achieve greater peripheral metabolic stress at the same absolute power and central cardiovascular stress. Moreover, SLEC is a form of small muscle mass exercise that can be used as a research tool in to study various topics. It can be used to evaluate the contribution of central and peripheral factors that influence fatigue (Elmer et al., 2013), as it can amplify the intensity of the small muscle mass without subsequent increase in whole body ${\dot{V}}_{O_{2}}$. It could also serve as a modality to study muscle sympathetic nerve activity and the contribution of the exercise pressor reflex during small muscle mass exercise (Stavres et al., 2020) due to the increase signalling that likely occurs from group III/IV afferents during SLEC. It can also be used to measure interlimb differences between dominant and non‐dominant legs (Iannetta et al., 2019), which could be a valuable tool to assess recovery following an injury. Additionally, future investigations should focus on the long‐term adaptations to SLEC including those with exercise intolerance due to metabolic and cardiovascular disease.

## AUTHOR CONTRIBUTIONS

B. Ryan Davis contributed to the study conception, design, data collection, analysis and drafting of the manuscript. Shane Aultman, Safwan Barnawi, Alex Sipolino contributed to the data collection and revisions of the manuscript. JM contributed to the conception, study design, manuscript revisions and advising. All authors have read and approved the final version of this manuscript and agree to be accountable for all aspects of the work in ensuring that questions related to the accuracy or integrity of any part of the work are appropriately investigated and resolved. All persons designated as authors qualify for authorship, and all those who qualify for authorship are listed.

## CONFLICT OF INTEREST

None declared.

## Data Availability

Data are available upon reasonable request.

## References

[eph70331-bib-0001] Abbiss, C. R. , Karagounis, L. G. , Laursen, P. B. , Peiffer, J. J. , Martin, D. T. , Hawley, J. A. , Fatehee, N. N. , & Martin, J. C. (2011). Single‐leg cycle training is superior to double‐leg cycling in improving the oxidative potential and metabolic profile of trained skeletal muscle. Journal of Applied Physiology, 110(5), 1248–1255. 21330612 10.1152/japplphysiol.01247.2010

[eph70331-bib-0002] Amann, M. , Blain, G. M. , Proctor, L. T. , Sebranek, J. J. , Pegelow, D. F. , & Dempsey, J. A. (2010). Group III and IV muscle afferents contribute to ventilatory and cardiovascular response to rhythmic exercise in humans. Journal of Applied Physiology, 109(4), 966–976. 20634355 10.1152/japplphysiol.00462.2010 PMC2963332

[eph70331-bib-0003] Burns, K. J. , Pollock, B. S. , LaScola, P. , & McDaniel, J. (2014). Cardiovascular responses to counterweighted single‐leg cycling: Implications for rehabilitation. European Journal of Applied Physiology, 114(5), 961–968. 24492992 10.1007/s00421-014-2830-0

[eph70331-bib-0004] Dela, F. , Handberg, A. , Mikines, K. J. , Vinten, J. , & Galbo, H. (1993). GLUT 4 and insulin receptor binding and kinase activity in trained human muscle. The Journal of Physiology, 469(1), 615–624. 8271219 10.1113/jphysiol.1993.sp019833 PMC1143890

[eph70331-bib-0005] dela Paz, G. N. , Walshe, T. E. , Leach, L. L. , Saint‐Geniez, M. , & D'Amore, P. A. (2012). Role of shear‐stress‐induced VEGF expression in endothelial cell survival. Journal of Cell Science, 125(4), 831–843. 22399811 10.1242/jcs.084301 PMC3311927

[eph70331-bib-0023] Dolmage, T. E. , & Goldstein, R. S. (2006). Response to one‐legged cycling in patients with COPD. Chest, 129(2), 325–332. 16478848 10.1378/chest.129.2.325

[eph70331-bib-0006] Dolmage, T. E. , & Goldstein, R. S. (2008). Effects of one‐legged exercise training of patients with COPD. Chest, 133(2), 370–376. 17925417 10.1378/chest.07-1423

[eph70331-bib-0007] Draper, S. , Kearney, S. G. , & McDaniel, J. (2019). Greater reliance on carbohydrates during single leg versus double leg cycling. Journal of Exercise and Nutrition, 2(2), 9.

[eph70331-bib-0008] Draper, S. , Singer, T. , Dulaney, C. , & McDaniel, J. (2022). Single leg cycling offsets reduced muscle oxygenation in hypoxic environments. International Journal of Environmental Research and Public Health, 19(15), 9139. 35897502 10.3390/ijerph19159139 PMC9331301

[eph70331-bib-0010] Elmer, S. J. , Amann, M. , McDaniel, J. , Martin, D. T. , & Martin, J. C. (2013). Fatigue is specific to working muscles: No cross‐over with single‐leg cycling in trained cyclists. European Journal of Applied Physiology, 113(2), 479–488. 22806085 10.1007/s00421-012-2455-0 PMC3934423

[eph70331-bib-0011] Elmer, S. J. , McDaniel, J. , & Martin, J. C. (2016). Biomechanics of counterweighted one‐legged cycling. Journal of Applied Biomechanics, 32(1), 78–85. 26398962 10.1123/jab.2014-0209

[eph70331-bib-0012] Evans, R. A. , Dolmage, T. E. , Mangovski‐Alzamora, S. , Romano, J. , O'Brien, L. , Brooks, D. , & Goldstein, R. S. (2015). One‐legged cycle training for chronic obstructive pulmonary disease. A pragmatic study of implementation to pulmonary rehabilitation. Annals of the American Thoracic Society, 12(10), 1490–1497. 26291542 10.1513/AnnalsATS.201504-231OC

[eph70331-bib-0013] Heidorn, C. E. , Elmer, S. J. , Wehmanen, K. W. , Martin, J. C. , & McDaniel, J. (2023). Single‐leg cycling to maintain and improve function in healthy and clinical populations. Frontiers in Physiology, 14, 1105772. 37187959 10.3389/fphys.2023.1105772 PMC10175616

[eph70331-bib-0014] Iannetta, D. , Passfield, L. , Qahtani, A. , MacInnis, M. J. , & Murias, J. M. (2019). Interlimb differences in parameters of aerobic function and local profiles of deoxygenation during double‐leg and counterweighted single‐leg cycling. American Journal of Physiology. Regulatory, Integrative and Comparative Physiology, 317(6), R840–R851. 31617749 10.1152/ajpregu.00164.2019 PMC6962629

[eph70331-bib-0015] Jeukendrup, A. E. , & Wallis, G. A. (2005). Measurement of substrate oxidation during exercise by means of gas exchange measurements. International Journal of Sports Medicine, 26(S1), S28–S37. 15702454 10.1055/s-2004-830512

[eph70331-bib-0016] LaScola, P. , Heidorn, C. E. , Pollock, B. , Burns, K. , & McDaniel, J. (2020). Physiological responses to counterweighted single‐leg cycling in older males. International Journal of Exercise Science, 13(2), 1487–1500. 33414863 10.70252/PCCP9259 PMC7745914

[eph70331-bib-0017] MacInnis, M. J. , Zacharewicz, E. , Martin, B. J. , Haikalis, M. E. , Skelly, L. E. , Tarnopolsky, M. A. , Murphy, R. M. , & Gibala, M. J. (2017). Superior mitochondrial adaptations in human skeletal muscle after interval compared to continuous single‐leg cycling matched for total work. The Journal of Physiology, 595(9), 2955–2968. 27396440 10.1113/JP272570 PMC5407978

[eph70331-bib-0019] Sidhu, S. K. , Weavil, J. C. , Rossman, M. J. , Jessop, J. E. , Bledsoe, A. D. , Buys, M. J. , Supiano, M. S. , Richardson, R. S. , & Amann, M. (2019). Exercise pressor reflex contributes to the cardiovascular abnormalities characterizing. Hypertension, 74(6), 1468–1475. 31607174 10.1161/HYPERTENSIONAHA.119.13366 PMC6854322

[eph70331-bib-0024] Staples, T. J. , Do‐Duc, A.‐A. , Link, J. E. , & Martin, J. C. (2020). Emphasizing one leg facilitates single‐leg training using standard cycling equipment. Scandinavian Journal of Medicine & Science in Sports, 30(6), 1017–1023. 32077131 10.1111/sms.13638

[eph70331-bib-0020] Stavres, J. , Luck, J. C. , Ducrocq, G. P. , Cauffman, A. E. , Pai, S. , & Sinoway, L. I. (2020). Central and peripheral modulation of exercise pressor reflex sensitivity after nonfatiguing work. American Journal of Physiology‐Regulatory, Integrative and Comparative Physiology, 319(5), R575–R583. 32877237 10.1152/ajpregu.00127.2020 PMC7789962

[eph70331-bib-0021] Wray, D. W. , Uberoi, A. , Lawrenson, L. , & Richardson, R. S. (2006). Evidence of preserved endothelial function and vascular plasticity with age. American Journal of Physiology. Heart and Circulatory Physiology, 290(3), H1271–H1277. 16272199 10.1152/ajpheart.00883.2005

